# Health Attitudes of Women Living in Religious Communities—A Preliminary Study

**DOI:** 10.3390/healthcare12191922

**Published:** 2024-09-25

**Authors:** Paulina Teodorczyk, Paweł Najechalski, Maciej Walędziak, Anna Różańska-Walędziak

**Affiliations:** 1 Clinical Nursing Department, Faculty of Medicine, Collegium Medicum, Cardinal Stefan Wyszynski University in Warsaw, 01-938 Warsaw, Poland; s.stellacsfn@gmail.com; 2 Faculty of Medicine, Lazarski University, 02-662 Warsaw, Poland; pawel.najechalski@lazarski.pl; 3 Department of General, Oncological, Metabolic and Thoracic Surgery, Military Institute of Medicine—National Research Institute, Szaserów 128 St., 04-141 Warsaw, Poland; 4 Department of Human Physiology and Pathophysiology, Faculty of Medicine, Collegium Medicum, Cardinal Stefan Wyszynski University in Warsaw, 01-938 Warsaw, Poland; aniaroza@tlen.pl

**Keywords:** religious communities, health-seeking attitudes, lifestyle, consecrated persons

## Abstract

Introduction: Members of consecrated life communities exhibit homogeneity with regard to factors that are relevant to health, including diet, daily activities, religiosity, and housing. This may be indicative of the manner in which the community influences the formation of the health-seeking attitudes of its members. Purpose of the study: The objective of this study was to validate a survey about health awareness and health-seeking attitudes among consecrated people and to identify potential issues to be improved. In addition, the study aspires to provide insights into the lifestyles of consecrated persons in Poland, based on the results of the survey and available data. Materials and Methods: The study group included 27 female participants, members of societies of apostolic life and non-habitual apostolic religious congregations of the Roman Catholic Church. The participants were invited to express their comments and suggestions on the survey, with the purpose of validating the survey before using it as a tool for a larger study. Results: All participants agreed that nutrition, rest, and physical activity had a significant impact on health. A total of 89% of participants considered their lifestyle as healthy and a similar proportion indicated that living in a consecrated community had a positive impact on their health, with the most positive influence of factors related to spiritual exercise, rhythm of the day, and sense of interpersonal connection within the community. Approximately 44% of participants indicated that their attitude was based on a healthy diet and adequate sleep quality. However, they identified an existing need to improve the balance between work and rest. Additionally, 4% of respondents admitted irregular eating habits, ignoring quality of alimentary products, non-satisfactory rest time, and insufficient sleep. Conclusions: Even though the purpose of this study was only to validate a survey predestined for a larger study, it already gives an insight into the level of awareness of health behavior and lifestyle of residents of religious communities.

## 1. Introduction

People living in communities of consecrated life are a homogeneous group in terms of lifestyle and values, in which religiousness, community life, and rhythm of the day play an important role and are based on the practice of the Evangelical Counsels of chastity, poverty, and obedience. The mission of an institute is shaped by a charism enshrined in the Constitutions, which condition a specific interpretation for each institute, setting it apart from the others [1]. Participants of this study are members of the Roman Catholic Church. Although consecrated persons are a minority both in the general society—0.04% in 2023—and in the Church community, they are a large group of 15,632 people. The number of consecrated persons in Poland has decreased by 25% since 1997, by 33% in the case of women, when compared to 2000 [2]. Members of religious communities constitute a group similar in terms of health-relevant factors such as diet, daily activities, religiousness, and housing conditions.

In 1983, the Code of Canon Law abolished the historical distinction between “orders” and “congregations”, unifying the nomenclature to the phrase “Institutes of religious life”. The forms of consecrated life include apostolic religious life, institutes devoted to contemplation, societies of apostolic life, secular institutes, and individual forms [1,3,4].

**Figure 1:**
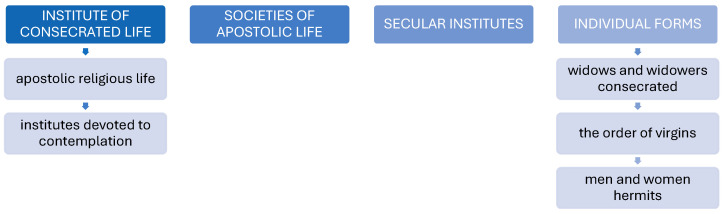
Forms of consecrated life.

The forms of consecrated life are presented in Figure 1.

The study of the lifestyles of consecrated persons and the influence of the community on their lifestyles is a novel contribution to the field. The uniqueness of our research is that the study was conducted among consecrated persons, which is uncommon despite the potential for such research to provide an exemplary illustration of the influence of collective lifestyle on the individuals. The reasons for this are limited access to consecrated religious communities and minimal involvement of these individuals in medical research. Consequently, we considered presenting the results of our research conducted in even a small group of participants significant and beneficial, as the topic has been insufficiently addressed in the existing literature and represents a new contribution to the field. Additional benefits of the study are the opportunity to evaluate potential strategies for enhancing the quality of life of these individuals, and the possibility of investigating the influence of the religious community, in which they reside, on their health attitudes. There is limited data in the literature on the subject of health attitudes and lifestyle of consecrated persons, which may be related to difficulties in acquiring data, due to lack of access to religious communities and a lack of willingness to participate in medical research [5]. The available data suggest that living in religious communities has a positive influence on mental health and immunity mechanisms when compared to the general population. Some studies also suggest that increased religiosity and spirituality may have a beneficial effect on the process of aging [6,7].

### Purpose of the Study

The objective of this study was to validate a survey about health awareness and health-seeking attitudes among consecrated persons and to identify potential issues to be improved. In addition, the study aspires to provide insights into the lifestyles of consecrated persons in Poland, based on the results of the survey and available data.

## 2. Materials and Methods

The study group included 27 female participants, members of societies of apostolic life and non-habitual apostolic religious congregations of the Roman Catholic Church. The mean age of the participants was 37.5 years (range 28 to 58), the mean age at which they entered the community was 24.0 years, and the mean length of their stay in the institute was 6.5 years. The majority of participants currently resided in Poland, with one individual of Ukrainian nationality who resided in Ukraine.

The survey comprised three sections. The initial section of the survey included questions about the respondents’ personal details and consisted primarily of open-ended questions. The second section of the survey comprised multiple-choice questions about physical activity, alimentary habits, rest, and health behavior. The final section of the survey was intended to assess participants’ beliefs and attitudes, with a particular focus on the impact of individual elements of community life on both psychological and physical well-being.

The participants were invited to express their comments and suggestions on the survey, with the purpose of validating the survey before using it as a tool for a larger study.

### Ethical Considerations

This study was anonymous and performed in accordance with the ethical standards laid out in the 1964 Declaration of Helsinki and its later amendments (Fortaleza). Participants were informed about the aim of this study and informed consent was obtained from every participant. The approval from the Bioethics Committee of the National Medical Institute of the Ministry of the Interior and Administration in Warsaw, Poland, with code 25/2024 was obtained on 19 April 2024.

## 3. Results

The daily physical activity indicated by nearly half of the participants as most frequent was related to household chores and professional work and the least frequent was leisure activity, chosen by only 3 participants. Nearly half of the respondents declared that they had to maintain a sedentary posture at work every day or at least once a week.

**Figure 2:**
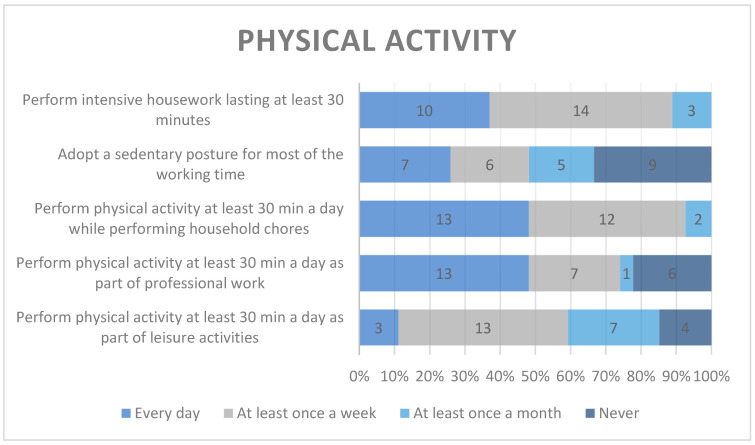
Physical activity.

The physical activity of the participants is presented in Figure 2.

Nearly all respondents reported eating regularly, with 3–5 meals daily, and drinking 1.5 L of liquids daily. More than half of respondents indicated eating fruits and vegetables at least once a week. More than 25% of the respondents declared eating animal-derived fats and highly processed alimentary products every day and almost 50% almost at least once a week. Approximately 22.2% of participants consumed sweets and/or sweetened beverages every day and 25.9% denied consuming any sugar-containing products. Almost all participants declared drinking coffee every day. Approximately 44.4% of the respondents practiced fasting at least once a month and 40.7% at least once a week, whereas 48.1% declared eating to improve their mood at least once a week, 33.3% at least once a month, and 11.1% stated “never”.

**Figure 3:**
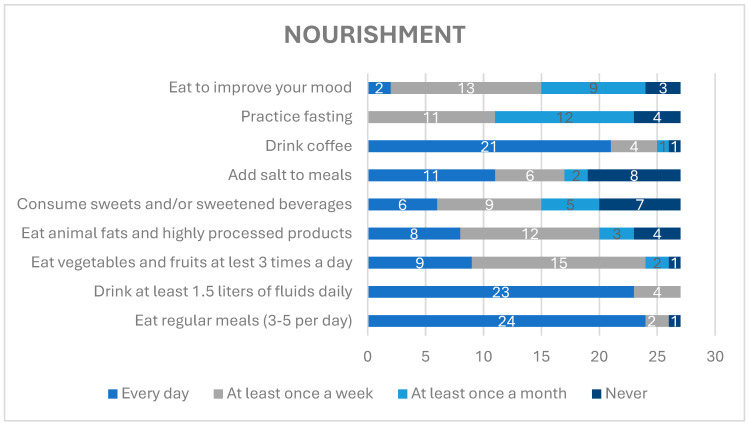
Alimentary habits.

Alimentary habits of the participants are presented in Figure 3.

The majority of participants stated that they had satisfactory sleep time every day or at least once a week. Almost the same number of participants indicated that they had difficulties with coping with stress and all participants stated that they experienced lowered or depressive mood, with the most frequent answer being “at least once a year”.

**Figure 4:**
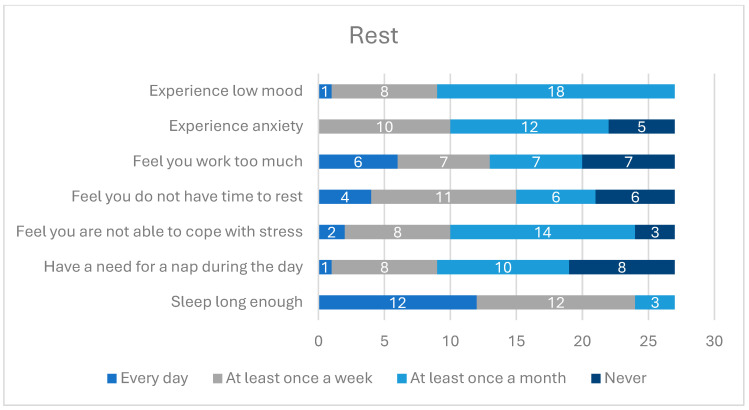
Rest time in religious communities.

Detailed data about the rest time of the participants is presented in Figure 4.

Approximately 40.7% of the respondents declared that they had a chronic disease. The majority of the respondents declared regular consumption of dietary supplements.

The distribution of taking dietary supplements and medications, including sleep medications, is presented in Table 1.

All participants were aware that nutrition, rest, and physical activity had an important impact on health. Approximately 89% of participants considered their lifestyle as healthy and thought that living in a religious community had a positive impact on their health, whereas 11% thought it had no influence. Approximately 44% of participants indicated that their health attitude was based on a healthy diet and adequate sleep quality, however, they felt the need to improve the balance between work and rest. Approximately 30% identified healthy nutrition, adequate balance between rest and work, regular physical activity, and good sleep quality as important for their health. Approximately 15% were aware that a balanced diet, regular meals, and physical activity should be the basis of their lifestyle; however, they indicated not getting enough sleep or rest. Approximately 7% did not get enough sleep, but they did take care of proper rest, including physical activity. Approximately 4% admitted not having regular meals, consuming food not always of good quality, not having time for rest, including physical activity daily, and not having enough sleep.

**Figure 5:**
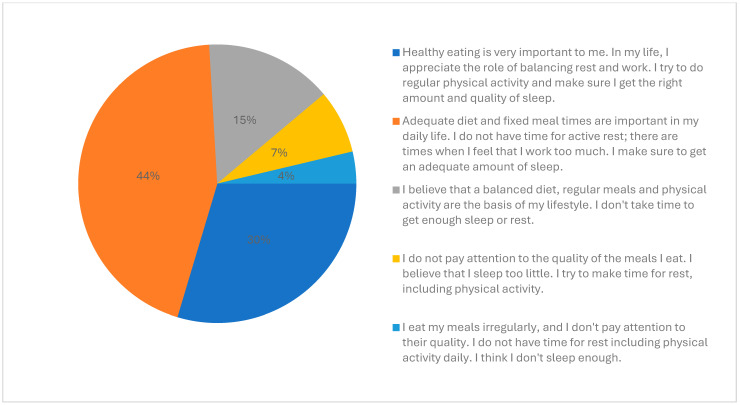
Main patterns of health attitudes of the participants.

The main patterns of health attitudes of the participants are presented in Figure 5.

The majority (59%) of respondents indicated that no aspect of community life has a deleterious effect on their health. No notable differences were observed between those who perceived community life as beneficial to health and those who did not across the age groups. Among individuals under 50 years of age, 84% concurred with this assertion, while 78% of those aged 50 and older expressed a similar view.

The majority of the respondents considered all of the mentioned elements of community life as having positive or rather positive effects on their psychological and physical well-being, with “Sense of connection in the community”; “Relationships in the community”; and “Community rhythm of the day” indicated as having a most positive impact on their health.

**Figure 6:**
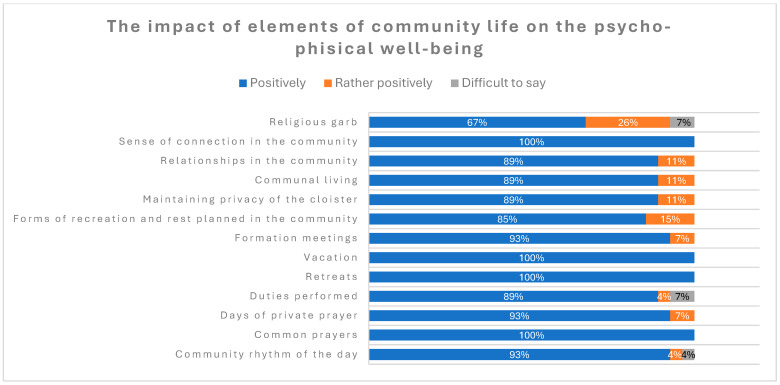
The impact of elements of community life on psychological and physical well-being.

The distribution of answers is presented in Figure 6.

“Relationships in the community” were most often indicated as having the most negative impact on the psychological and physical health of the participants. It is noteworthy that this factor is referenced as both one of the most positive and one of the most negative. The majority of survey participants indicated that it has a notable impact on health outcomes.

**Figure 7:**
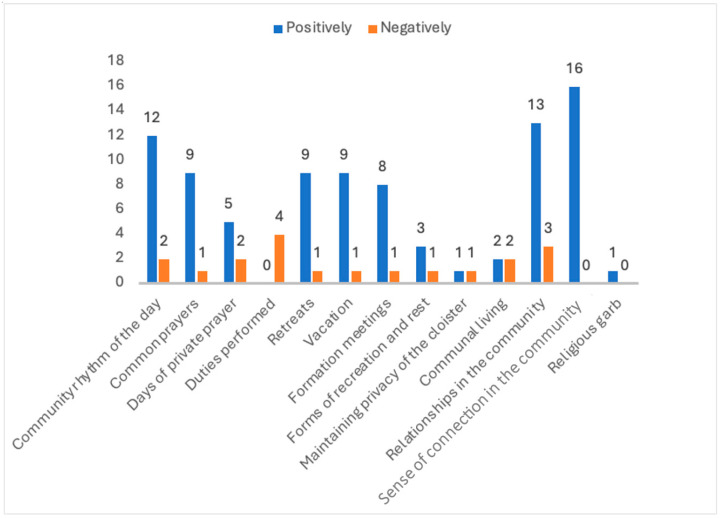
The number of answers about the positive and negative impact on different elements of psychological and physical health.

The distribution of answers about the positive and negative impact of different elements of community life is presented in Figure 7.

The respondents were invited to express their comments and suggestions on the survey. The most frequent requests were to expand the range of responses in the second part of the questionnaire, by including an option to select “several times a year” as an answer. There were also suggestions about some stylistic amendments.

## 4. Discussion

The existing research on health behavior and health-related attitudes among members of religious communities is scarce and inconclusive, which may be related to the low accessibility of this group and low confidence of the consecrated persons in participating in medical research [5]. The available data, though very limited, mostly show the positive influence of living in religious communities on both psychological and physical health, especially with respect to the process of aging [8]. In our study, 89% of the participants considered living in a religious community as having a positive effect on their health, and the same rate declared their lifestyle as being healthy. Even though we conducted our preliminary research with a small group of participants, the results appear to be interesting and indicate a need for a larger study. All participants agreed that nutrition, rest, and physical activity had a significant impact on health. A total of 89% of participants considered their lifestyle as healthy and a similar proportion indicated that living in a consecrated community had a positive impact on their health, with the most positive influence being factors related to spiritual exercise, rhythm of the day, and sense of interpersonal connection within the community. Approximately 44% of participants indicated that their attitude was based on a healthy diet and adequate sleep quality. Many of the participants found the community cohesion and support they received along with a sense of connection with the community beneficial, such as group prayer or formation meetings, of great positive influence on their health. However, interpersonal relationships were found by some of the participants to be a primary source of stress.

WHO (World Health Organization) guidelines for physical activity state that the recommended level of physical activity for adults and elderly people is 150–300 min per week [9]. More than half of members of religious communities participating in our study declared spending 30 min a day performing physical activity while working or carrying out household chores, and only 11% indicated performing physical activity as a form of leisure activity. The SHARE study (Survey of Health, Ageing and Retirement in Europe) showed that only 36.4% of Polish people had high physical activity at least once a week and only 34.2% had moderate activity at least once a week [10]. This difference can be related to the age of the participants—in the SHARE study, the participants were older than 55 years of age.

In a study by Nowak et al., a conclusion similar to our results can be found. Nowak et al. showed that in Poland, people often consider physical activity performed during professional work as sufficient and an adequate substitute for recreational sports in their leisure time. However, professional physical activity cannot be considered a positive element of a healthy lifestyle, because, in most cases, it overstrains the body in an asymmetric direction. Additionally, the same study showed that students in fields related to mental health and spirituality (e.g., theology) had a lower level of physical activity than students whose fields of study were related to health and physical activity [11].

Schoot and Krull stated in their study that 30% of consecrated persons performed physical exercise once or twice a week for at least 30 min [12]. In our study, nearly 50% of participants declared that they adopted a sedentary posture for most of their working time at least once a week, including 23% who admitted doing so every day. These results can be compared to the results presented by Biernat and Piotrowska. They showed in their study that people of working age in Poland work in a sedentary posture for more than 32.6 h a week and 13.6% do so for more than 7.5 h a day. Both studies showed that “white-collar” workers adopted a sedentary posture more often than “blue-collar” workers during their working hours, whereas this difference did not exist during non-working hours [13].

In a study on eating habits conducted in Poland and Greece, 50.2% of Polish participants responded that they regularly ate 4–5 meals a day and 21.1% drank the recommended amount of at least 1.5 L of liquids, with even better results in religious communities—89% declared that they ate regularly and 85% drank 1.5 L or more of liquids a day. The results in the religious communities were less favorable than in the general Polish population with respect to the daily intake of fruit and vegetables, 33% vs. 67.6%, respectively [14].

In our research, 56% of respondents declared that they ate to improve their mood at least once a week. Research conducted on a group of Taiwanese adolescents showed that emotional eating was one of the most important risk factors for increased consumption of unhealthy food [15]. This was confirmed in our research—62% of participants declared that they eat to improve their mood at least once a week and consumed animal fats and highly processed products, sweets, and/or sweetened beverages daily or at least once a week.

Coffee, next to water and tea, is one of the most commonly consumed drinks around the world. In Poland, average coffee consumption is 1–2 cups of coffee a day. Consumption of coffee has increased in the last 10 years, and now, it involves over 80% of the general adult Polish population, which is parallel to our study, where daily coffee consumption was admitted by 77% of respondents [16].

A study of the relationship between spirituality and mental health, which included 70 priests and 70 nuns from apostolic and religious congregations, found that involvement in spirituality and prayer improves mental health and reduces the risk of mental disorders [8]. A high level of spirituality, as expressed in religious practices, was found to result in more effective stress management strategies and a greater sense of self-fulfillment, even in the elderly population [6,8]. In our study, we also observed that factors related to common prayers, community rhythm of the day, and sense of connection in the community were declared by the participants as having the most positive impact on their health. The majority of participants did not report daily occurrence of anxiety symptoms. Approximately 33% of participants reported experiencing these symptoms at least once a week, 4% experienced low mood daily, and the remainder experienced it rarely. These findings are more favorable than those observed in the adult population in Poland surveyed in 2022 during the COVID-19 pandemic, where the levels of anxiety, stress, depression, and sleep disorders were 43.5%, 61.3%, 50.4%, and 44.7%, respectively [17]. Approximately 10–20% of the general adult population suffer from insomnia [18]. In our study, 11% of participants declared that they slept long enough more rarely than once a month and 15% admitted taking sleep medications on a regular basis.

### Limitations of the Study

The main limitation of this study is the small sample of participants in the survey and the specificity of the study group, which included only female participants, members of different institutes of consecrated life.

In order to ensure an appropriate number of participants, it is necessary to consider the number of members of female congregations in Poland. Based on the assumption that this number is 15,632, the number of participants should be 375. In consideration of the fact that the primary study would be conducted at an international scale, the requisite number of participants for a *p*-value of 0.05 and a 95% confidence interval for the global population of nuns in 2023, which is estimated to be 608,958, is 384. It is anticipated that a greater number of female participants will be involved in the main study.

## 5. Conclusions

The conclusions of our study are only preliminary, due to the small validation sample size, as the survey was part of preparation for a larger study. However, it already provides an interesting insight into the awareness of health behavior and lifestyle of people living in religious communities. As declared by the majority of participants, living in a religious community can have a positive impact on psychological and physical health, and is associated with having a healthy lifestyle and health-seeking behavior in terms of regular meals and hydration. There is also a high level of awareness of the need for rest and adequate sleep. The main elements of community life that are considered as having the most positive impact on the awareness of health-promoting attitudes are those related to prayer, the rhythm of the day, and a sense of stability.

## Figures and Tables

**Table 1:** Dietary supplements and medication consumption.

	Yes	No
Have a chronic illness	11	16
Take dietary supplements regularly	12	15
Take non-prescription medications regularly	5	22
Permanently take medications prescribed by a doctor	8	19
Take sleep medications	4	23

## Data Availability

The original contributions presented in the study are included in the article, further inquiries can be directed to the corresponding author.
